# How older adults experience the age-friendliness of Skopje: Results of the validation of the AFCCQ for use in North Macedonia and a representative survey

**DOI:** 10.1016/j.heliyon.2024.e30372

**Published:** 2024-04-27

**Authors:** Daniel Pavlovski, Jeroen Dikken, Elisabeta Bajrami Ollogu, Joost van Hoof

**Affiliations:** a Mother Teresa University – Skopje, Faculty of Social Science, Skopje, Macedonia; b Research Group of Urban Ageing, Faculty of Social Work & Education, The Hague University of Applied Sciences, Johanna Westerdijkplein 75, 2521, EN Den Haag, the Netherlands; c Faculty of Health, Nutrition & Sport, The Hague University of Applied Sciences, Johanna Westerdijkplein 75, 2521, EN Den Haag, the Netherlands; d Department of Systems Research, Faculty of Spatial Management and Landscape Architecture, Wrocław University of Environmental and Life Sciences, ul. Grunwaldzka 55, 50-357, Wrocław, Poland

**Keywords:** Age-friendly cities, Quantitative, Evaluation, Assessment, North Macedonia

## Abstract

Hundreds of cities and communities in the world have joined the WHO's Global Network for Age-Friendly Cities and Communities since 2010. In order to do quantitative assessments of the age-friendliness of cities, the Age-Friendly Cities and Communities Questionnaire (AFCCQ) was developed for the Dutch municipality of The Hague. The purpose of this study was first to translate and test the validity and reliability of the AFCCQ for use in North Macedonia and second to explore perceptions on age-friendliness of the bicultural and bilingual City of Skopje. The AFCCQ proved valid for use in North Macedonia. Overall, older adults in Skopje experience the age-friendliness of the city as neutral (in seven out of nine domains). The best score (“slightly satisfied”) was found in the domain of Housing domain, which was rated positive in all ten municipalities. The lowest total score (“slightly dissatisfied”) was found in the domain of Outdoor spaces and buildings scoring, which received negative scores in eight out ten municipalities. In five out of nine domains differences were observed between the Albanian and Macedonian communities. The Albanian sample has slightly higher scores in two domains: 1) Housing and 2) Civic Participation and Employment, while the Macedonian sample scored higher in three domains: 1) Communication and Information; 2) Outdoor Spaces and Buildings and 3) Transportation. A hierarchical cluster analysis further revealed the presence of six distinct age-friendly typologies that can be used for a better understanding of subpopulations in the city and draft policies and action programs on the city level.

## 1 Introduction

According to the World Bank [1], more than 50 % of the global population currently resides in urban areas. By 2045, this urban population is projected to increase by 1.5 times, reaching 6 billion people. The rapid pace and magnitude of urbanization present numerous challenges, including meeting the escalating demand for affordable housing, viable infrastructure such as transportation systems, basic services, and employment opportunities. These challenges are particularly significant for the nearly 1 billion urban poor who reside in informal settlements near potential sources of income.

To address the needs of older citizens and create cities that are better attuned to their requirements, the World Health Organization (WHO) launched the Age-Friendly Cities and Communities worldwide program in 2007 [2]. In 2010, the WHO established the Global Network for Age-friendly Cities and Communities to connect cities, communities, and organizations globally with the shared goal of creating environments that are conducive to ageing well [3]. This initiative, which acknowledges the effects of global population ageing and rapid urbanization, focuses on local-level actions that facilitate the full participation of older adults in community life and promote healthy and active ageing. The WHO Age-friendly Cities framework, outlined in the Global Age-friendly Cities Guide, proposes eight interconnected domains that help identify and address barriers to the well-being and involvement of older adults [3].

The country of North Macedonia, which is situated in the south-east of Europe, has no active members in the global network, as the participation of cities and communities in Central and Eastern Europe is still quite limited [[4], [5], [6]]. Nevertheless, the country is taking measures for improving the lives of older adults. In order to tackle the health and social challenges of the ageing population, the Ministry of Labor and Social Policy adopted the National Strategy for Older People 2010–2020 [7]. Three key priorities were identified in this strategy, namely the improvement of the system for social protection for older people; the development and strengthening of the health care system for the older people; and the integration of the older people in social life. In 2014, Ministry of Health introduced several initiatives to provide greater access to health services for older people, such as home visits by community nurses (also called patronage nurses), the rural doctor's project and mobile pharmacies [8]. In the field of institutional social protection for older people, the government of North Macedonia is implementing a set of measures for support the private investors to open nursing homes in the country. According to the data of the Ministry of Labor and Social Policy [9] in June 2023, there are 40 private institutions for non-family social protection of older people (nursing homes) in North Macedonia, of which 29 are located in the city of Skopje and its surroundings. In the area of non-institutional social protection in the city of Skopje, there are three daycare centers for older people, in the municipalities of Centar, Čair and Saraj. Also, in nine out of ten municipalities in the city of Skopje, there are centers for care and help of older people at home. Through the Law on Social Security for Older People [10], the state provides financial support to the older people who are not pension beneficiaries and have no income. Also, through the social protection system, the state provides to the older people different types of support in type of cash transfers and/or services [11]. In the field of transportation, the government provides free rides with Macedonian railways for all older people in the country one weekend a month and a free city transport for all pensioners in Skopje four days a week. However, although the central and local authorities take various measures to improve the quality of life of older people, there is still a lack of strategic (medium/long-term) document for older people at the state level. At present, North Macedonia lacks a coordinated approach at the state and municipal level that will synthesize, prioritize and coordinate policies and measures for active and healthy ageing.

The current study provides a first attempt to systematically investigate the age-friendliness in North Macedonia, using the Age-friendly City and Communities Questionnaire (AFCCQ) [12]. The research question for this study focuses on how older adults residing in Skopje experience the age-friendliness of their city. Considering this research question, we hypothesize that there are variations in the perception of age-friendliness among older adults living in different municipalities in Skopje, with lower scores expected for Šuto Orizari and Saraj. Furthermore, we assume that older adults with diverse ethnic backgrounds may exhibit different scores due to cultural values and traditions, within the political reality of a single country. In this regard, North Macedonia may serve as an example for the increasingly multi-ethnic cities in Western Europe, North America and other parts of the world.

## 2 Methodology

### 2.1 Profile of the City of Skopje

The Republic of North Macedonia is located in Southeast Europe. More concretely it is one of Western Balkan countries with a total area of 25,436 km^2^. In the past, the country was part of Yugoslavia, from which it declared independence in 1991. In the entire territory of the Republic of North Macedonia and in its international relations, the official language is Macedonian, which is written, in the Cyrillic script. Another language spoken by at least 20 % of the citizens is Albanian, which is the second official language in the country.

In terms of ethnicity, 58.4 % are Macedonians, 24.3 % Albanians, 3.9 % Turks, 2.5 % Roma and 14.8 % others. The average age of the population in 2021 was 40.8 years; 39.9 for males and 41.7 for females. The life expectancy is 76.34 years (74.39 for males and 78.29 for females). The number of older people in 2002 was 213,712 or 10.60 % of the total population, while in 2021 it was 315,331 or 17.17 %. This means that the population of North Macedonia is ageing rapidly.

According to the 2021 census 526.502 inhabitants live in the City of Skopje, that is, 28.67 % of the total population in the country (Table 1) [13]. The total number of citizens over the age of 65 living in the City of Skopje is 90,983 (17.28 %) (Table 1). The ratio between the sexes is 48.67 % (39.748) for males and 51.33 % (51.235) for females. From the numbers for the citizens in the City of Skopje, it can be seen that some municipalities have a higher total number of people compare to other and also there are differences in the number and the percentage of citizens aged 65 years and over (Table 1).

**Table 1:** Total population and number/percentage of people aged 65 years and over.

	Total population	Age 65+	Age 65+ [%]
**North Macedonia**	1.836,713	315,331	17.17
**Skopje**	526,502	90,983	17.28
1. Aerodrom	77,735	15,756	20.26
2. Butel	37,968	6163	16.23
3. Gazi Baba	69,626	12,508	17.96
4. Gorče Petrov	44,844	8799	19.62
5. Karpoš	63,760	12,874	20.19
6. Kisela Voda	61,965	12,101	19.52
7. Saraj	38,399	3307	8.61
8. Centar	43,893	10,111	23.03
9. Čair	62,586	7328	11.71
10. Šuto Orizari	25,726	2036	7.91

**Fig. 1:**
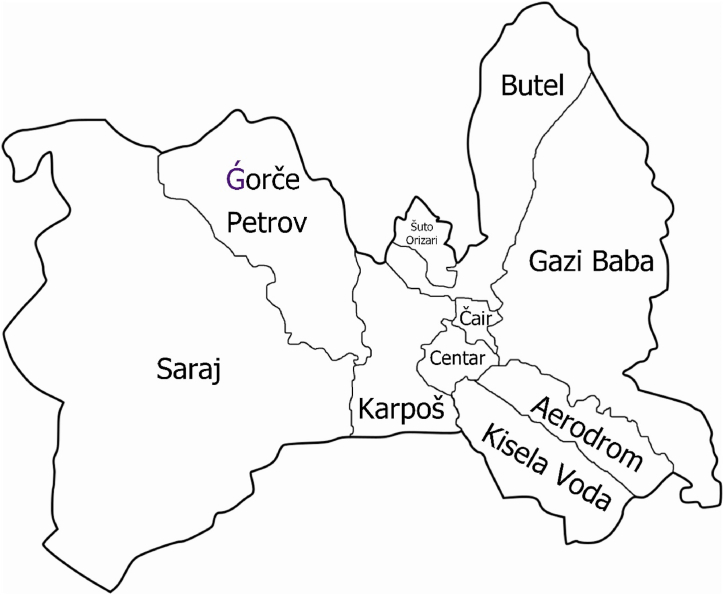
Map of the ten municipalities of the City of Skopje.

The City of Skopje is the capital and largest city of North Macedonia, which is also considered the administrative, political, economic, cultural and educational-scientific center of the country. It is located in the northern part of the country, lying alongside the Vardar River. The City of Skopje, as a separate unit of local self-government, is composed of ten municipalities: Aerodrom, Butel, Gazi Baba, Gorče Petrov, Karpoš, Kisela Voda, Saraj, Centar, Čair and Šuto Orizari (Fig. 1). The total area of the City of Skopje is 1818 km^2^ and the largest municipality in the City of Skopje is Saraj with an area of 229 km^2^, while the smallest is Čair with a surface area of only 3.5 km^2^.

At the level of the city of Skopje, the population distribution according to ethnicity is similar to the situation at a national level. However, regarding the municipalities that are part of the city of Skopje, there are certain significant differences (Table 2).

**Table 2:** Ethnic composition of the municipalities of the City of Skopje.

Census 2021	Nationality			
	Macedonians	Albanians	Roma	Other
**North Macedonia**	58.4 %	24.3 %	2.5 %	14.8 %
**Skopje**	58.7 %	22.8 %	3.5 %	15.0 %
1. Aerodrom	85.2 %	1.1 %	0.6 %	13.1 %
2. Butel	44.8 %	37.1 %	1.3 %	16.8 %
3. Gazi Baba	65.0 %	20.3 %	2.8 %	12.0 %
4. Gorče Petrov	80.2 %	3.9 %	2.4 %	13.5 %
5. Karpoš	80.9 %	3.6 %	0.9 %	14.6 %
6. Kisela Voda	84.0 %	0.7 %	0.8 %	14.5 %
7. Saraj	2.6 %	90.1 %	0.7 %	6.6 %
8. Centar	76.8 %	2.5 %	1.4 %	19.3 %
9. Čair	8.6 %	67.4 %	2.1 %	21,9 %
10. Šuto Orizari	3.5 %	34.3 %	43.8 %	18.4 %

The data show that out of a total of ten municipalities in the City of Skopje, six municipalities (Aerodrom, Gazi Baba, Gorče Petrov, Karpoš, Kisela Voda and Centar) have a population that is characterized by a dominance of ethnic Macedonians while in two municipalities (Saraj and Čair) the Albanian population predominates. In one municipality (Šuto Orizari) ethnic Roma are the majority, and in another municipality (Butel) the ratio between the percentage of ethnic Macedonians and Albanians is approximately the same (Table 2).

### 2.2 Design

**Fig. 2:**
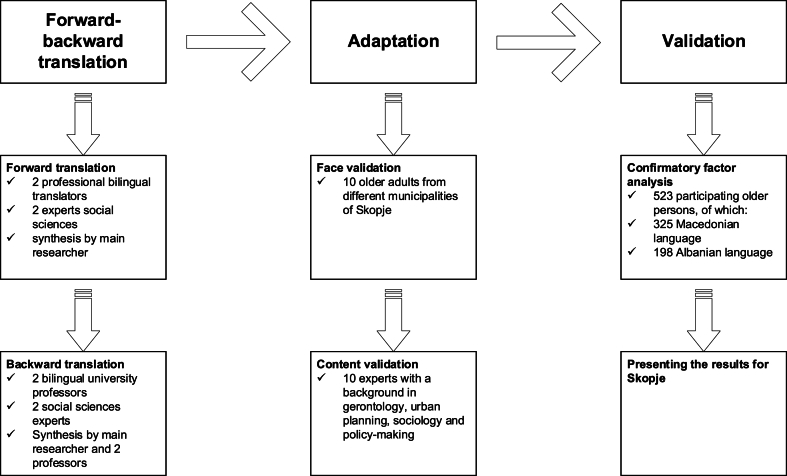
Process of translation, adaptation and validation of the AFCCQ-MK and AFCCQ-AL.

The study followed a cross-sectional design. This design followed the intended use of the Age-Friendly Cities and Community Questionnaire (AFCCQ) as a single self-assessment measurement at one point in time to determine how older adults in Skopje perceive the age-friendliness of their city and neighborhoods [12]. The scale consists of 23 items and covers 9 domains of age-friendliness, respectively: 1) Housing; 2) Social participation; 3) Respect and social inclusion; 4) Civic participation and employment; 5) Communication and information; 6) Community support and health services; 7) Outdoor spaces and buildings; 8) Transportation and 9) Financial situation. The scores are expressed on a 5-point Likert scale. The higher a score on the domains the more age-friendly a city is considered to be. Fig. 2 shows the process of translation, adaption and validation of the AFCCQ for North Macedonia.

### 2.3 Translation, adaptation and validation of the AFCCQ

The British English version of the AFCCQ [12] (including instructions and response format) was forward-translated to Macedonian and Albanian languages by four translators. Two of them were professional translators (licensed and legally authorized as translators) and with many years of work experience, and the two were experts with a background in social sciences with an excellent knowledge of spoken and written English, whose mother language is either Macedonian or Albanian. The translators were bilingual (i.e., fluent in both English and the Macedonian/Albanian languages). For synthesis, the two forward-translated versions of the AFCCQ (AFCCQ-MK and AFCCQ-AL) and the original AFCCQ-EN version were then compared by the principal researcher regarding ambiguities and discrepancies of words, sentences and meanings, which were then discussed and resolved by the research team. This process led to the preliminary initially translated version of the instruments. Next, the AFCCQ-MK and AFCCQ-AL were translated back into the English language by two university professors of English language and literature employed at Mother Teresa University in Skopje and by two other experts with an academic background in social sciences and with an excellent knowledge of spoken and written English.

These translators were completely blind to the AFCCQ-EN (i.e., they have never read nor seen the original AFCCQ-EN) and produced a back-translated version of the AFCCQ, resulting in two back-translated versions of the AFCCQ in English. For synthesis, the instructions, items and response template of the two back-translations were again compared by the main researcher and two professors of social work and social policy. Any ambiguities and discrepancies regarding the cultural meaning and colloquialisms or idioms in words and sentences of the instructions; the items; the response format between the two back-translations; and between each one of the two back-translations and the original AFCCQ-EN were again discussed and resolved through a process of reaching consensus by the research team. The results can be found in Appendix A, Appendix A for the Macedonian and Albanian versions. The original, version in English can be found in Appendix 3.

To assess adaptation of the translated AFCCQ, face validation was done by involving ten older adults, who reside in different municipalities of the City of Skopje; five of them were of Macedonian nationality for the Macedonian language questionnaire, and five of Albanian nationality for the Albanian language questionnaire. Content validation was done with ten experts who live and work in the Republic of North Macedonia, of which six are of ethnic Macedonian background and four are of ethnic Albanian background. The face and content validity were assessed using the Item Content Validity Index (I-CVI). I-CVI is defined as the proportion of older adults/experts who rate the content as relevant/valid for their respected city and/or culture. Older adults were contacted in person explaining that they have to rate the relevance of the AFCCQ items on a four-point Likert scale (1 = not relevant, 2 = somewhat relevant, 3 = quite relevant, 4 = highly relevant). They had to be able to read and understand their respected languages and diversity in educational level was sought. A panel of experts were contacted in person independently appraise the relevance of the items with respect to the construct, study population (older adults), and purpose for the City of Skopje on the same four-point Likert scale. For both groups, the I-CVI was calculated for each of the 23 items by I-CVI = Nr/N where Nr = number of people voting [3] or [4] and N = total number of recruited people. Lynn [14] and Polit et al. [15] described an item to be excellent when the I-CVI value was greater than 0.78. Data of all steps were analyzed using SPSS version 29.0 (IBM Corp., 2020). Finally, both groups were asked whether the items covered the entire construct (i.e., are there any themes or items missing that you consider relevant to measure age-friendliness in your city?).

After translation and testing cultural adaptation and validity, a Confirmatory Factor Analysis (CFA) was carried out to assess construct validity using the model described by Dikken et al. [12]. In addition, the Measurement Invariance (MI) was assessed between the Macedonian and Albanian language versions to test whether comparisons could be made (see for a detailed statistical description on CFA and MI, Appendix 4 [[16], [17], [18], [19], [20]]). If the measurement invariance (MI) is demonstrated then the participants across all groups interpret the individual items, as well as the underlying latent factor, in the same way and the Macedonian and Albanian language groups in Skopje can be compared on scores on age-friendliness, enhancing interpretation for the city. To evaluate the fit of the models, several fit indices were considered. First, the normed χ^2^ which is less sensitive to sample size than the χ^2^, with values up to 5 to evaluate model fit to be adequate [21]. Second, the Comparative Fit Index (CFI) and Tucker Lewis Index (TLI) which should be 0.9 or greater [22]. The Root-Mean Squared Residual (SRMR) should be less than 0.08 [22] and the Root-Mean Square Error of Approximation (RMSEA) should be less than 0.08 for moderate fit [23]. Finally, Internal consistency of the model was evaluated using composite reliability which should be above 0.70 to be considered appropriate for reliability [24]. CFA analyses were done using IBM SPSS Amos version 28.0 (IBM Corp., 2021).

### 2.4 Recruitment and participants

After face and content validity was assessed, recruitment of individuals over 65 years of age who live in the city of Skopje started for psychometric validation (CFA) and to assess city results. Based on the data of the census conducted in 2021 [13], participants were recruited from each of the ten municipalities in the City of Skopje. Three criteria for inclusion in the research were used: 1) targeted were individuals of 65 years old or above 2) older adults who live in home conditions (not residing in institutions) and 3) older adults who can communicate in Macedonian or Albanian language.

In order for the sample to be representative, the included respondents had to reflect the demographic characteristics of the population over the age of 65 in the City of Skopje. The ratio between males and females had to reflect the state of the municipality in which they live. In addition, the participants had to be from different parts of the municipality in which they live, for the reason that although the older adults are not equally distributed in all parts of the municipalities, there are still older adults in all parts. In terms of age, the respondents are divided into three age groups: 65–69, 70–74 and 75+. In terms of nationality, respondents included in the survey had to reflect the ethnic picture obtained in the 2021 census. Therefore, this study made use of a sample in which all neighborhoods are represented (Table 3).

**Table 3:** Demographics of participants Skopje, North Macedonia (total = 523).

	Skopje total (n = 523)	Macedonian language (n = 325)	Albanian language (n = 198)	Sig. difference
**Sex**				0.030
Male	230 (44 %)	131 (40.3 %)	99 (50.0 %)	
Female	293 (56 %)	194(59.7 %)	99 (50.0 %)	
**Age,** Mean (SD)	71.9 (6.43)	72.3 (5.43)	71.3 (7.76)	0.29
65–69	193 (36.9 %)	110 (33.8 %)	83 (41.9 %)	
70–74	172 (32.9 %)	117 (36.0 %)	55 (27.8 %)	
75+	158 (30.2 %)	98 (30.2 %)	60 (30.3 %)	
**Educational level**				**<0.001**
ISCED 0–2	196 (37.5 %)	40 (12.3 %)	156 (78.8 %)	
ISCED 3–4	224 (42.8 %)	186 (57.2 %)	38 (19.2 %)	
ISCED 5–6	100 (19.1 %)	98 (30.2 %)	3 (1.5 %)	
ISCED 7–8	3 (0.6 %)	1 (0.3 %)	1 (0.5 %)	
**Years living in Skopje,** Mean (SD)	60.0 (16.28)	63.0 (13.74)	55.1 (19.78)	**<**0**.001**
**Aerodrom**^2^	59 (11.3 %)	58 (98.3 %)	1 (1.7 %)	**<**0**.001**
**Butel**^2^	49 (9.4 %)	19 (38.8 %)	30 (61.2 %)	**<**0**.001**
**Gazi Baba**^2^	74 (14.1 %)	39 (52.7 %)	35 (47.3 %)	**<**0**.001**
**Ǵorče Petrov**^2^	35 (6.7 %)	35 (100 %)	0 (0 %)	**<**0**.001**
**Karpoš**^2^	65 (12.4 %)	62 (95.4 %)	3 (4.6 %)	**<**0**.001**
**Kisela Voda**^2^	48 (9.2 %)	46 (95.8 %)	2 (4.2 %)	**<**0**.001**
**Saraj**^2^	31 (5.9 %)	0 (0 %)	31 (100 %)	**<**0**.001**
**Centar**^2^	36 (6.9 %)	35 (97.2 %)	1 (2.8 %)	**<**0**.001**
**Čair**^2^	97 (18.5 %)	8 (8.2 %)	89 (91.8 %)	**<**0**.001**
**Šuto Orizari**^2^	29 (5.5 %)	23 79.3(%)	6 (20.7 %)	**<**0**.001**
**Type of dwelling**				**0.005**
Owner-occupant	506 (96.7 %)	320 (98.5 %)	186 (93.9 %)	
Private rent	17 (3.3 %)	5(1.5 %)	12 (6.1 %)	
**Living together with a spouse or partner**	438 (83.7 %)	253 (77.8 %)	185 (93.4 %)	**<0.001**
**Receiving care**	67 (12.8 %)	47 (14.5 %)	20 (10.1 %)	0.416
**Living with one or more chronic conditions**	229 (43.8 %)	136 (41.8 %)	93 (47.0 %)	0.215
**Using a wheeled walker or a wheelchair**	77 (14.7 %)	28 (8.6 %)	49 (24.7 %)	**<0.001**
**Self-rated Quality of Life,** Mean (SD) (score 1 very low – 10 very high)	5.94 (3.82)	6.19 (4.74)	5.53 (1.20)	**0.017**

The data were collected through direct contact (interviews) with the participants. For the Macedonian speaking sample (total 325), 199 interviews were made by the organization CRPM* (Center for Research and Policy Making, a non-profit organization that is performing policy research and analysis, adult education and public policy advocacy) and 126 by the research team from Mother Teresa University. The research team conducted the interviews with members of the “University of Third Age” and older users of services at day care centers for the older adults in the municipalities of Centar, Čair and Saraj. Students of the Mother Teresa University in Skopje of the Social Work and Social Policy program of the Faculty of Social Sciences collected the data in the Albanian language (total 198). The main criteria for the selection of the students were to be fluent in the Albanian language, to have an overall grade higher than eight (of out ten) and to live in (and know well) the municipality in which the data were to be collected. The selected students received a one-day training course how to approach and communicate with older adults, as well as the technique of explanation the questions and filling out the questionnaire. The training was conducted by the members of the research team from Mother Teresa University.

### 2.5 Age-friendly typologies in the city of Skopje

A total 523 older adults participated in this study (325 Macedonian speaking and 198 Albanian speaking), none having missing values on AFCCQ-MK and AFCCQ-AL items. Besides the AFCCQ questionnaire, older adults reported information related to demographic characteristics (Table 3). First, mean scores for the city, its districts and between Macedonian and Albanian speaking older adults were examined. Then, in order to develop age-friendly typologies, cluster analysis was used to group similar study participants based on the nine domains of the Age-Friendly Cities and Communities Questionnaire (AFCCQ) [[25], [26], [27]]. First, the Likert scale data from the AFCCQ were normalized by dividing the scores of the domains by the number of items used for each domain. Then, the number of clusters were determined using a hierarchical cluster analysis (HCA) in which Ward's method was selected to assess association and similarity [[28], [29], [30]]. To validate the number of clusters, the study sample was divided into separate samples (Macedonian speaking and Albanian speaking), and cluster analysis was repeated. After identifying the most stable number of clusters, *k*-means cluster analysis was performed for clarification and interpretation. The percentage breakdown of demographic characteristics within each cluster were examined to identify salient features. Categories that represented 75–99 % were considered "highly likely" for a particular cluster, while categories falling between 51 and 74 % were deemed "likely." If no category exceeded 50 %, it either indicated that the category was not salient for that feature or, if appropriate, categories were combined. These analyses were done using SPSS version 29.0 (IBM Corp., 2020).

### 2.6 Research ethics

Certification of ethical acceptability for research involving human subjects was obtained from the Head of Quality Assurance and Management Office at the Mother Teresa University in Skopje on January 13, 2023 (certificate number 03–29/1). The study was conducted in accordance with the principles of the Helsinki Declaration of Human Rights. The purpose of the study was explained to the individuals participating in the study. Participants consented to their participation by filling out the survey or being interviewed.

## 3 Results

### 3.1 AFCCQ translation, adaptation and validation

During the initial translation from English to Macedonian and Albanian, some minor adjustments were made by the research team to ensure a consistent use of language in the final versions. The back translation closely resembled the original items, although there were a few instances where the translation was not an exact fit or a loose translation was used. After reaching consensus by the research team, it was determined that no further changes were needed in the final versions of the back translation.

To assess the face validity of the AFCCQ-MK and AFCCQ-AL, a group of ten older individuals (8 males, 2 females, age ranging from 67 to 77 years) participated. For content validation 10 academic experts in the field of social work and/or gerontology (2 males and 8 females) evaluated the relevance and readability of the 23 questionnaire items. Both older adults (face validity) and scientific experts (content validity) assessed all items of the AFCCQ relevant for both the Macedonian and Albanian cultures. Whilst doing so, they did not provide any comments or suggestions to improving the readability of the items.

The confirmatory factor analysis (CFA) model for the original-factor structure of the AFCCQ demonstrated a reasonable fit to the data, as indicated by the results presented in Table 4. The normed χ^2^value was 3.560, indicating an adequate fit. Additionally, the robust comparative fit index (CFI) and Tucker-Lewis index (TLI) values were 0.899 and 0.869 respectively, which almost surpassed the threshold of 0.9 [22]. The root mean square error of approximation (RMSEA) value was 0.070, which is considered adequate [23]. The robust standardized root mean square residual (SRMR) value was 0.0742, slightly below the recommended threshold of 0.08 [31]. In addition, the estimated covariance paths between the factors were all below the 0.85 cut-off, indicating sufficient discriminant validity, thus confirming that the items measure distinct yet related factors.

**Table 4:** Fit of data from Skopje with the original model as described by Dikken et al. [12].

Model	Normed χ^2^*(DF)*	Comparative Fit Index (CFI)	Tucker Lewis Index (TLI)	Root-Mean Squared Residual (SRMR)	Root-Mean Square Error of Approximation (RMSEA)
Skopje total Model 1.	3.560 *(195)*	0.899	0.869	0.0742	0.070 *(*0*.064 -* 0*.076)*
Albanian language Model 1. (n = 198)	2.434 *(195)*	0.861	0.819	0.0739	0.085 *(*0*.076 -* 0*.095)*
Macedonian language Model 1. (n = 325)	3.077 *(195)*	0.877	0.840	0.0577	0.080 *(*0*.073 -* 0*.087)*

To assess the internal consistency of the model derived from the final CFA, the composite reliability was examined (Table 5). The results indicate that all but one factor surpass the threshold of >0.70, indicating a reasonable reliability. The factor Civic participation and Employment in particular scored lower for the AFCCQ-AL, most likely because a large part of the ethnic Albanian population over the age of 65 in North Macedonia, especially women, are not formally employed.

**Table 5:** Composite reliability per factor of the AFCCQ-MK and AFCCQ-AL.

	Housing	Social Participation	Respect and Social Inclusion	Civic Participation and Employment	Communication and Information	Community Support and Health Services	Outdoor Spaces and Buildings	Transportation	Financial Situation
AFCCQ-**AL**	0.856	0.80	0.812	**0.498**	0.869	0.765	0.770	0.836	0.820
AFCCQ-**MK**	0.889	0.75	0.875	**0.688**	0.868	0.851	0.846	0.886	0.910

In order to compare groups having different backgrounds in the City of Skopje in a valid way, the AFCCQ-MK and AFCCQ-AL should measure identical constructs with the same structure across the different groups. To assess this, Measurement Invariance was tested (Table 6). Full metric invariance was established, as demonstrated by the trade-off between model fit and model complexity, which did not significantly worsen compared to the Configural model (Metric model: ΔCFI = 0.003; ΔRMSEA = 0.000; ΔSRMR = 0.0022). Partial scalar invariance was established after the intercept of item 3, 4 (social Participation) and item 13 (Community support and health services) were unconstrained (Partial scalar model: ΔCFI = 0.001; ΔRMSEA = 0.001; ΔSRMR = 0.0008). On these three items some cultural differences exist. Despite partial scalar invariance of these three items, the latent mean scores can still be compared adequately as literature describes that full scalar invariance is not necessary to make substantive analysis, provided that at least two items are invariant [16,17].

**Table 6:** Measurement invariance between the ethnic Macedonian and Albanian samples in Skopje.

	χ^2^	Df	Δχ^2^	Sig.	CFI	ΔCFI	RMSEA	Δ RMSEA	SRMR	Δ SRMR	TLI
Configural	1071.831	388	–		0.871	–	0.058	–	0.0756	–	0.831
Metric	1097.385	402	25.554	<0.000	0.868	0.003	0.058	0.000	0.0734	−0.0022	0.879
Scalar	1177.775	416	80.39	<0.000	0.856	0.012	0.059	0.001	0.0746	0.0012	0.825
Partial scalar^a^	1118.581	413	21.196	<0.000	0.867	0.001	0.057	0.001	0.0742	0.0008	0.836

a Relaxing intercept item 3, 4 (SP) and 13 (CSHS), **Δ** fit indices are compared with the metric model.

### 3.2 The measured age-friendliness of the City of Skopje

#### 3.2.1 AFCCQ scores for the City of Skopje

Overall, older adults in Skopje experience the age-friendliness of the city in “neutral” rank. The total score on the AFCCQ is 7.32 ± 12.29 on a scale of −46 to +46 (Table 7 and its footnote for the interpretation of the scores). Analysis according the various AFCCQ domains shows that Skopje scores a “neutral” in seven out of nine domains. The best score (“slightly satisfied”) is in the *Housing* domain with total score 2.48 (on a scale of −4 to +4), and positive scores in all ten municipalities. The lowest total score (“slightly dissatisfied”) is in the *Outdoor spaces and buildings* domain with score −0.50 (on a scale of −4 to +4) with negative scores in eight out ten municipalities.

**Table 7:**
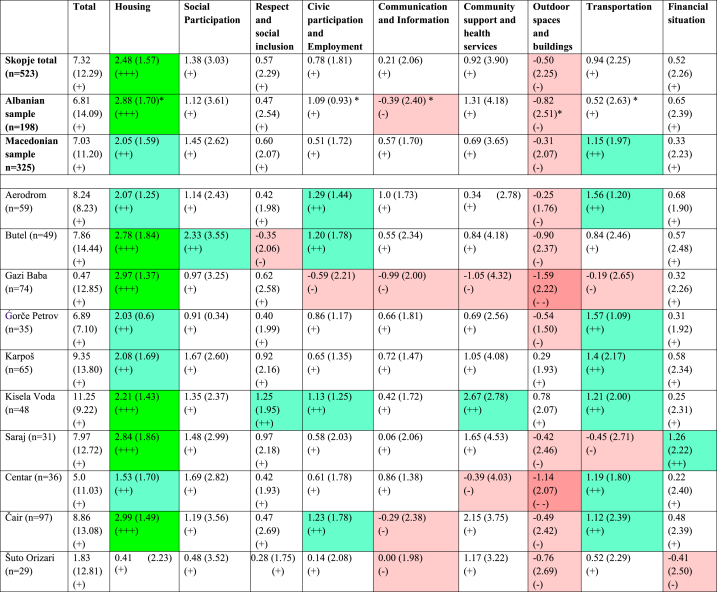
Scores (Mean + - SD) for AFCCQ domains for the municipality of Skopje and its districts (n = number of participants). Regression of Sociodemographic on the AFCCQ (Total and 9 Domains). Shown are coefficients and standard errors in parentheses.

The colored zones represent how dissatisfied or satisfied older adults are regarding the city as a whole or a specific domain. Scores in green zones mean that people are neutral to slightly satisfied (+, white) to very satisfied (++++, dark green), Scores in red mean that people are slightly dissatisfied (-, light red) to very dissatisfied (- - - -, dark red) based on the interpretation and presentation method presented by Dikken et al.

(2020). *= significant difference between Albanian and Macedonian samples tested by the independent sample t-test

#### 3.2.2 AFCCQ scores for the municipalities

The overall scores for each of the ten municipalities in the City of Skopje, which were created in 2004, shows that some scores higher than others, but all overall scores are in the “neutral’ rang. The municipality Kisela Voda has the highest total score of 11.25 ± 9.22 (with no negative scores in any domain), whilst Gazi Baba the lowest 0.47 ± 12.85 (with negative scores in five out nine domains).

Scores for the various domains of the AFCCQ scale shows that *Housing* domain scores high in the municipalities Butel, Gazi Baba, Saraj and Čair, with a lowest score in Šuto Orizari. The *Social participation* domain scores in "neutral" rank in nine municipalities. Regarding the *Respect and social inclusion* domain, the municipality of Kisela Voda has highest score and Butel lowest, other eight municipalities are in “neutral” rank. In the *Civic participation and employment* domain three municipalities have scores in “slightly satisfied” and in the *Communication and information* domain the scores are relatively low in all municipalities. In the *Community* support *and health services* domain, the municipality of Kisela Voda has highest score, followed by municipality of Čair, whilst Gazi Baba and Centar have negative scores in “slightly dissatisfied” rank.

The scores in *Outdoor spaces and buildings* domain are negative in eight municipalities and the lowest compared to the other domains. In the *Transportation* domain, six municipalities have scores in rank "slightly satisfied" followed by two municipalities in the "neutral" rank, whilst two municipalities (Gazi Baba and Saraj) have scores in “slightly dissatisfied” rank. The *Financial situation* domain, scores relatively low in the most of the municipalities. In this domain best score has municipality Saraj and the lowest Šuto Orizari.

The total scores in the AFCCQ domains shows significant differences between the Albanian and Macedonian samples in five out of nine domains (Table 7). The Albanian sample has slightly better scores in two domains: 1) *Housing* and 2) *Civic Participation and Employment*, while the Macedonian sample has higher scores in three domains: 1) *Communication and Information*; 2) *Outdoor Spaces and Buildings* and 3) *Transportation*.

### 3.3 Age friendly typologies in the city of Skopje

**Fig. 3:**
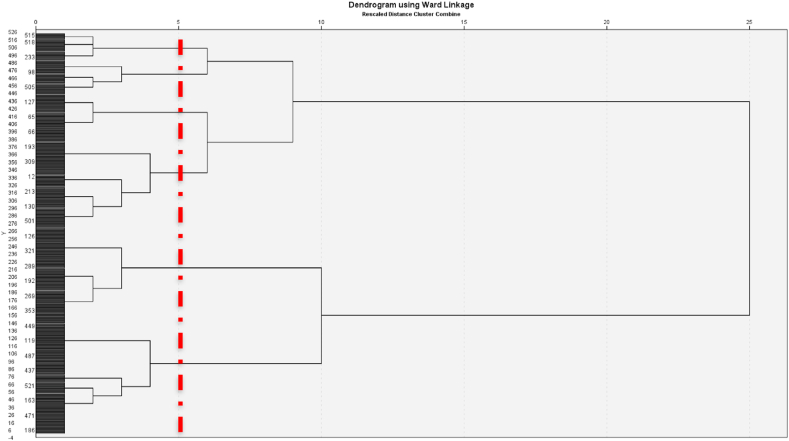
Dendrogram using Ward's method revealing six stable clusters.

The hierarchical cluster analysis generated a dendrogram that revealed the presence of six distinct and stable clusters that represent typologies (Fig. 3). By employing *k*-means clustering, it was determined that significant differences (<0.001) existed among all clusters across the nine domains of the AFCCQ. Typology 1, consisting of 71 individuals, reported the least favorable levels of age-friendliness in Skopje, encompassing the majority of the subdomains. *Housing* and *Respect and social inclusion* domains received high scores in Typology 2 (72 individuals), but the other domains of the AFCCQ obtained lower scores. Typology 3 (77 individuals) achieved average scores across all AFCCQ domains, except for their financial situation, which received the lowest score compared to other typologies. Typology 4 (52 individuals) exhibited lower scores in the *Transport* and *Financial situation* domains. While Typology 5 (134 individuals) generally displayed positive outcomes, it recorded the lowest score in the *Respect and social inclusion* domain. Lastly, Typology 6 (117 individuals) demonstrated the highest levels of age-friendliness across all domains, indicating a particularly optimistic perspective on Skopje's age-friendliness.

Based on the distinctive characteristics observed in each cluster (Table 8), narratives for each of the age-friendly typologies were developed (Table 9). In terms of personal factors, the average age across typologies did not differ significantly, with Typology 6 being the youngest (mean age: 71.1 ± 6.3 years) and Typology 1 being the oldest (mean age: 74.4 ± 6.6 years). Female representation was the highest in Typology 5 (67.9 %). Typology 2 consist a significant number (61.1 %) of individuals with lower education scores (ISCED 0–3) and Typology 3 had large percentage of individuals with medium education (59.7 %). However, Typology 6 had a notably larger percentage of individuals with higher education (28.2 %) compared to other typologies. Typology 3 had a higher percentage of Macedonian speaking individuals (76.6 %) compared to other typologies.

**Table 8:**
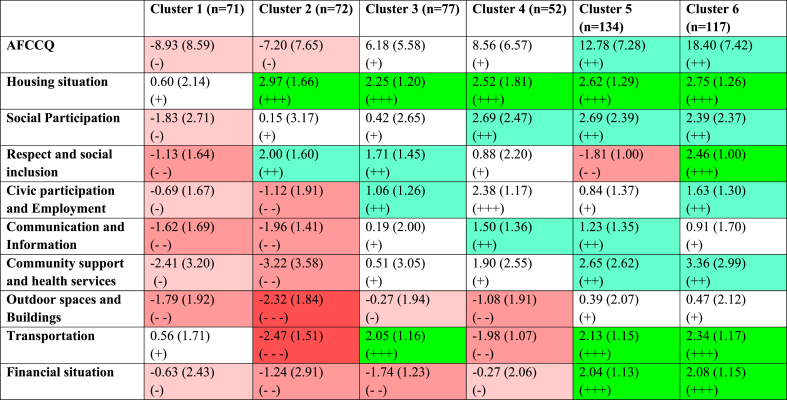
Cluster scores on the AFCCQ domains.

**Table 9:** The six age-friendly typologies based on most salient demographics.

	Typology 1 (n = 71)	Typology 2 (n = 72)	Typology 3 (n = 77)	Typology 4 (n = 52)	Typology 5 (n = 134)	Typology 6 (n = 117)
Personal factors	74.4 ± 6.6 years old, living in Skopje for a long time (mean 62 ± 17.3 years).	71.4 ± 9.1 years old, living in Skopje for a long time (mean 61.2 ± 17.0 years).	71.2 ± 4.9 years old, living in Skopje for a long time (mean 58.4 ± 15.6 years).	73.0 ± 7.1 years old, living in Skopje for a long time (mean 61.2 ± 17.7 years).	71.7 ± 4.7 years old, living in Skopje for a long time (mean 61 ± 15.1 years).	71.1 ± 6.3 years old, living in Skopje for a long time (mean 57.5 ± 16.3 years).
	Likely to be Macedonian speaking* (60.6 %)	Likely to be Albanian speaking* (54.2 %)	Likely to be Macedonian speaking* (76.6 %)	Equal number Albanian/Macedonian speaking (50.0 %)	Likely to be Macedonian speaking* (70.1 %)	Likely to be Macedonian speaking* (59.8 %)
	Likely to be female* (56.6 %).	Likely to be female* (55.6 %).	Likely to be male* (55.8 %).	Likely to be female* (59.6 %).	Likely to be female* (67.9 %).	Likely to be male* (53.8 %).
	Likely to have completed a low level of education (ISCED 0–2)* (54.9 %), with 29.6 % of people medium- and 15.5 % highly educated.	Likely to have completed a low level of education (ISCED 0–2)* (61.1 %), with 36.1 % of people medium- and 2.8 % highly educated.	Likely to have completed a medium level of education (ISCED 3–4)* (59.7 %), with 27.3 % of people low- and 13.0 % highly educated.	42.3 % completed a low level of education (ISCED 0–2), with 34.6 % of people medium- and 23.1 % highly educated.	47.8 % completed a medium level of education (ISCED 3–4), with 26.1 % of people low- and 26.1 % highly educated.	41.9 % completed a medium level of education (ISCED 3–4), and 28.2 % a high education (ISCED 5–8), with 29.9 % of people low educated.
Housing situation	Highly likely to be owner occupant** (95.8 %)	Highly likely to be owner occupant** (97.2 %)	Highly likely to be owner occupant** (94.8 %)	Highly likely to be owner occupant** (100 %)	Highly likely to be owner occupant** (97.0 %)	Highly likely to be owner occupant** (96.6 %)
	Highly likely to live together** (85.9 %).	Highly likely to live together** (88.9 %).	Highly likely to live together** (84.4 %).	Highly likely to live together** (86.5 %).	Highly likely to live together** (79.9 %).	Highly likely to live together** (82.1 %).
	People mainly live in Šuto Orizari (19.7 %); Čair (18.3 %); Gazi Baba (16.9 %).	People mainly live in Gazi Baba (38.9 %); and Čair (18.1 %).	People mainly live in Aerodrom (18.2 %); Čair (18.2 %); Karpoš (18.2 %).	People mainly live in Čair (17.3 %); Butel (13.5 %); Karpoš (11.5 %); Centar (11.5 %); and Saraj (11.5 %).	People mainly live in Čair (20.1 %); Karpoš (14.2 %); Gazi Baba (13.4 %); and Butel (12.7 %).	People mainly live in Čair (17.9 %); Kisela Voda (15.4 %); Aerodrom (14.5 %); and Karpoš (12.8 %).
Health	Likely to have at least one chronic condition* (54.1 %), 11.3 % received some sort of healthcare, and 25.4 % used a mobility aid.	37.5 % of the people have a chronic condition, only 1.4 % received some sort of healthcare, and 29.2 % used a mobility aid.	Likely to have at least one chronic condition* (50.6 %), 13.0 % received some sort of healthcare, and 6.5 % used a mobility aid.	48.1 % of the people have at least one chronic condition, 17.3 % received some sort of healthcare, and 19.2 % used a mobility aid.	35.1 % of the people have at least one chronic condition, 11.2 % received some sort of healthcare, and 9.0 % used a mobility aid.	Likely to have at least one chronic condition* (45.1 %), 11.3 % received some sort of healthcare, and 25.4 % used a mobility aid.
Quality of life	Mean score QoL 5.13 ± 1.74	Mean score QoL 5.21 ± 1.74	Mean score QoL 5.42 ± 1.95	Mean score QoL 5.71 ± 1.92	Mean score QoL 7.38 ± 6.64	Mean score QoL 5.68 ± 1.97
Age friendly domain of interest	Respect and social inclusion; Communication and information; Outdoor spaces and buildings	Outdoor spaces and buildings; Transportation	Financial situation; Outdoor spaces and buildings	Outdoor spaces and buildings; Transportation	Respect and social inclusion	Outdoor spaces and buildings

**highly likely >75 %, *Likely 51–74 %, no salience <50 %.

In terms of where older adults live, Typology 3 had a significantly higher percentage (38.9 %) of people living in Gazi Baba municipality. In Typology 1, a larger percentage (19.7 %) of people live in the municipality of Šuto Orizari. The older citizens of Čair municipality are relatively equally distributed in all six typologies.

Regarding housing situations, all six typologies primarily consisted of homeowners, living together. Regarding one's health status, Typology 1 had the highest proportion of individuals with at least one chronic condition (54.1 %) and reported the lowest quality of life rating (5.13 ± 1.74). Typology 3 also had a relatively high prevalence of chronic conditions (50.6 %). Typology 4 had a larger number of individuals receiving care (17.3 %), whilst Typology 2 had larger number of individuals using mobility aids (29.2 %). Typology 2 had the lowest number of individuals receiving care (1.4 %). The highest scores for quality of life were found among the people of Typology 5 (7.38 ± 6.64).

## 4 Discussion and implications

The aim of this study was to examine how older adults experience the age-friendliness of the city of Skopje. According to the outcomes of the cross-culturally validated AFCCQ for use in North Macedonia for the two language groups, the study finds that older adults experience the age-friendliness of their city in a rather neutral way. Skopje scores a neutral score in seven domains, “slightly dissatisfied” in the domain of *Outdoor spaces and buildings* domain and only one “satisfied” in the *Housing* domain. These scores are lower than, for instance, scores from a similar survey conducted in the Netherlands [32], where older adults residing in the municipality of The Hague experienced the age-friendliness of their city as satisfactory in all AFCCQ domains.

Our study revealed the presence of six distinct and stable age-friendly clusters (i.e., typologies). Cluster 1 demonstrates the lowest, while Cluster 6 the highest levels of age-friendliness in all domains. In Cluster 1 the majority of older adults are women (56.6 %) and have completed a low level of education (ISCED 0–2) (54.9 %), also Cluster 1 had the highest proportion of individuals with at least one chronic condition (54.1 %) and reported the lowest quality of life rating (5.13 ± 1.74), whilst in Cluster 6 the majority are men (53.8 %) and have completed a medium level of education (ISCED 3–4) (41.9 %). With each cluster, going from one to six, the perceived age-friendliness of the city increases. This goes hand in hand with a trend in increasing education levels, perceived health, and self-reported quality of life. There appears to be a strong correlation among these demographic factors and the perceived age-friendliness of the city, consistent with earlier findings demonstrated by van Hoof et al. [27,32] in the Netherlands and Ivan et al. [33] in Romania. In this study, this trend is not observed to correlate with age, even though age is often associated with various socio-economic factors such as poverty, illness, and lower quality of life [34]. The extent of this association can vary depending on numerous factors including access to healthcare, social support systems, economic opportunities, and individual circumstances which in this study proved more important in rating the perceived age-friendliness of their city. Therefore, addressing disparities in access to resources and opportunities is crucial across all age demographics to promote overall well-being and equity in the city of Skopje.

### 4.1 Findings of the AFCCQ

The lowest overall score in the domain of *Outdoor spaces and buildings* (with an even lower score for the Albanian sample), and the fact that none of the municipalities have a positive result in this domain, shows that the infrastructure in both the urban and rural parts of the City of Skopje is not adapted to the needs of the older adults. These results correspond with those reported by van Hoof et al. [32,35] namely, the *Outdoor spaces and building* domain score relatively low in various districts in the municipality of The Hague. Open spaces are a common venue for older adults in their daily lives, particularly in dense urban cities, which provide small living spaces [36]. By engaging in outdoor recreation, older adults have the potential to meet the needs related to meaning, purpose and social connectedness [37,38]. When analyzing the situation on the municipal level, the present study showed that lowest score in this domain was found for the municipality of Gazi Baba. A possible explanation is that the physically separated neighborhoods in this municipality (mainly because of industrial and business facilities) and the existence of a large number of rural settlements with poor infrastructure make it difficult for older adults to move around easily. In contrast to our expectations, the municipality of Centar also has a low score in this domain. The possible explanation lies in the fact that this municipality occupies the central area of the capital of the Republic of North Macedonia where the parliament and the government are located, and it is also the administrative, health, cultural, educational and business center of the country with a lot of people and traffic on a daily basis, which creates challenges for the older adults, including busy traffic and noise.

Another domain with a low overall score is *Communication and information,* with lower scores found among the Albanian sample. In this domain, three municipalities have negative results: Gazi Baba (with a predominant ethnic Macedonian population and a Macedonian mayor), Čair (with a predominant ethnic Albanian population and an Albanian mayor) and Šuto Orizari (with a dominant ethnic Roma population and a Roma mayor). According to these results, the majority of the older citizens of Skopje, regardless of their ethnicity, and the fact that they live in a municipality where the official language is their mother tongue, are not satisfied with the communication and information provided by the central and local authorities. This can be related to lower scores on the self-reported educational level of Albanian speaking participants, but then again, the results of our study are also in line with the results of an Australian study by Everingham et al. [39], who found that many older adults report that they are not well-informed and, presumably as a result, not well connected with their community. The research by Everingham and colleagues identifies both a range of barriers for older adults to accessing information and also their preferences regarding information provision [39]. It is important for policymakers to improve their way of communicating and informing older people, because this ensures that information is effectively understood and accessible to all older people regardless of their educational backgrounds, fostering clearer communication and informed decision-making. According Age UK London [40] the ability to receive timely and practical information to help manage life and meet personal needs is vital for active ageing. In addition, it is important that older citizens are able to stay connected with people and events to avoid the difficulties posed by social isolation. The way that the physical environment and public buildings are designed and maintained has a major impact on the mobility, independence and quality of life of older people and the extent to which they can age in place [41]. The results from our study are in line with the results of another study conducted in North Macedonia by Totikj et al. [42], who found that the older adults in the city of Skopje are not sufficiently informed about their rights and services that they can use. The older adults would like to expand the opportunities for social and health benefits. A number of obstacles in terms of access to services that appeared from the study by Totikj et al. [42] were a lack of information on existing services and about their rights. In addition, written materials were insufficiently available in the languages of their respective communities.

In the domain of *Community* support *and health services* seven municipalities scored neutral. The lowest score has the municipality of Gazi Baba, followed by the municipality of Centar. The situation in Gazi Baba is partly understandable due to the fragmentation of the municipality into the large number of settlements with a concentration of most of the service providers in the central area of the municipality and the lack of providers in other parts. The results from our study are in line with the results of Wu et al. [40], who found that older adults who had one or more general practitioner practices in their local areas were less likely to report dissatisfaction with health services than those who had none. However, in contrast to our expectations, which are based on the fact that there are a large number of public and private health facilities and social service providers in the municipality of Centar, the municipality has a negative result in this domain. An alternative explanation is that older citizens may have a problem with the accessibility and affordability of the services. According Quinlan et al. [43] and Casado et al. [44] numerous structural barriers including lack of awareness, availability, accessibility, and affordability influence older adults’ use of formal services. As the population ages in North Macedonia, the family structures are changing and the health and social systems have a limited capacity to provide integrated and person-centered care, leaving older people in the country to face a number of health, social and economic challenges [45]. Population ageing needs all service provisions and systems to be made more efficient, provide better support to families which are taking care for their older relatives (by adapting their work schedules, or compensating them for the caring time) and supporting family relationships [46]. The increase in the older population in North Macedonia has not been accompanied by an adequate increase in service provision; either social care or healthcare; public or private healthcare; and institution, community or home-based health care [47]. The data from Albania suggest that the public system of healthcare is unprepared to meet the increased needs of the older population [48]. Spending on social care services at the national and local level is very low compared to the ambitious goal set out in the law of providing a comprehensive set of services in line with the need of the population [49]. The current limited capacity of the public long-term care system implies that frail older adults have no alternative but to rely on their families and relatives. Although family support is essential in the provision of care and is strongly connected to the traditional Albanian culture, which relies predominantly on the traditional intra-family provision of long-term care and depends heavily on unpaid female carers, it is no longer considered to be a sustainable solution [50].

The domain of *Financial situation* also has a low score on the AFCCQ scale, with a “neutral” score in eight (out of ten) municipalities. In this domain, the municipality of Šuto Orizari has the lowest score, compare to other municipalities. The possible explanation is that the unemployment and the total rate of informal employment in this municipality are the highest compared to other municipalities in the City of Skopje. Also, a large part of the older citizens does not receive a pension because of the fact that in the earlier periods of their lives, they were not officially employed and so they do not meet the conditions for acquiring a pension. In contrast to this, the highest score in this domain was found in the municipality of Saraj. A possible explanation can be found in the predominant rural character of the municipality with its lower cost of living, compared to other municipalities, as well as the fact that part of the population there grow fruit and vegetables themselves for their own consumption, and a smaller part of the population produces such products even for trade motives. Disparities in old age in income and wealth, access to financial services and employment often reflect accumulated disadvantage due to one's location, sex, socio‐economic status and other characteristics, ageist attitudes and practices, and lacking or inadequate laws and policies or their enforcement that provide for equality and minimum standards of living [51]. Multiple social identities, race, class, gender, and sexual orientation shape economic and social experiences that accumulate over the life course and determine economic status in late life [52].

In the domain of *Transportation*, negative results in the “slightly dissatisfied” range are found in two municipalities (Gazi Baba and Saraj), having a higher proportion of Albanian speaking participants demonstrating significantly more health problems, i.e., the number of reported chronic diseases and the use of mobility aids, which may potentially be of influence. An additional explanation is that, on the one hand, these municipalities have poor infrastructure and a large number of neighborhoods physically separated from each other, and from the other hand, a small number of bus lines and bus stops. Staying connected to communities and social networks enables older adults to contribute and connect with society and is associated with positive mental and physical health, facilitating independence and physical activity while reducing social isolation [53].

In the domain of *Civic participation and employment*, four municipalities have result in “slightly satisfied”, five in “neutral” and one in “slightly dissatisfied” rank (with lower score of the Macedonian sample). A possible explanation is the fact that the majority of older Albanian people still live in “traditional patriarchal” families, where respect for the older adults is at a higher level compared to Macedonians who usually live in “modern” type of families. However, over time, the Albanian community may also face a decline in the level of respect for older adults.

The domain of *Social participation* scores as neutral with no significant differences between municipalities. In line with sores in this domain, is also a score in *Respect and social inclusion* domain, with neutral scores in eight municipalities, whilst one (Butel) has a “slightly dissatisfied” score. The results in these two domains show that the older citizens from different municipalities in Skopje and different ethnic background face similar challenges. Social participation is considered a modifiable determinant of health and well-being and has been proposed as a means to reduce this risk [54]. An age-friendly city should provide options for older adults to contribute to their communities, through paid employment or voluntary work.

The City of Skopje only has а positive score in the *Housing* domain. It is the highest score compared to all other domains on AFCCQ scale (with a higher score for the Albanian sample, possibly because of the significant difference in living alone or living together). In this domain, in five out of ten municipalities (Butel, Gazi Baba, Kisela Voda, Saraj and Čair), the older citizens are "satisfied" with the situation. Only in one municipality (Šuto Orizari) the results are lower because of the poor infrastructure, where the part of the population living in bad housing conditions and without basic utilities. According to the WHO [55] the housing conditions of older people are often linked to their quality of life and whether they are able to age independently and actively in their community. Appropriate housing design and its proximity to community and social services allow older residents to live comfortably and safely, while housing affordability gives them peace of mind. Home is an important determinant of well-being for older people. It can enable them to age in place improving their life and delaying institutionalization [56]. A study by Poland by Kazak et al. [57] has shown that accessibility of housing is of particular relevance to being able to age-in-place, and the items of the AFCCQ relate to this topic in particular. Affordability of energy and utilities [[58], [59], [60], [61], [62]] and the rent older people pay are not a part of this instrument. Even though the older adults are not faced with a lack of adequate housing, they may still have to cope with loneliness, which may affect their mental and physical health. The abandonment of the older adults by other family members, which means they now live alone, is a recent problem that the older adults are facing in Albania [48], and perhaps in ethnic Albanian communities in neighboring countries, too.

The results of this study show that older people in the multi-ethnic City of Skopje in general face the same challenges in their everyday life, despite their different ethnical and cultural background. Most of the reasons for the low scores on the AFCCQ scale are related to the policies and practices of central and local authorities. However, the results also show that in some domains there are differences between citizens from different ethnic groups, such as a lower educational level, worse financial situation and housing conditions among the Roma population compare to other ethnic groups. On the other hand, the family solidarity is higher among the Albanian population compare to other ethnic groups, but older Albanian females have a lower education level and are in a worse financial situation than Albanian males. The differences show that the City of Skopje should have general approach in addressing the needs of the older citizens in most of the AFCCQ domains, but in some domains, it should have an individual approach for each of the municipalities and ethnic groups. The City of Skopje can use the results of this study to identify the priorities of older adults, but also the domains where the more detailed research focused on the needs of the different ethnical subgroups is needed. Policy makers should give priority to the domain of *Outdoor spaces and buildings* as the data show that older people living in all ten municipalities of the City of Skopje are not satisfied with the current situation in this domain. The lower scores for the Albanian speaking sample show that the situation in the municipalities with a predominant ethnic Albanian population is worse compared to the others. A possible explanation is the fact that this population predominantly lives in high-density urban municipalities or rural parts of the city with poor infrastructure. Another domain that should be prioritized by policymakers is the domain of *Communication and information*. The lower scores for the Albanian speaking sample indicate that local authorities in municipalities with a predominant Albanian population should develop effective strategies and implement measures for an adequate and timely provision of information to older adults about topics which are relevant to this age group.

In the future, it would be of great interest to compare the data gathered in Skopje with data collected in other Eastern European countries, Balkan countries and even Turkey, as the AFCCQ has also been validated for use in Turkey by Özer et al. [63] and Romania by Ivan et al. [33]. Also, it would be of great interest to study the ageing populations in Western European cities who are becoming increasingly diverse from an ethnic, cultural and/or religious perspective [64,65,20]. The findings from Skopje may provide the first insights in what such diversity could mean in practice.

### 4.2 Strengths and limitations

One of the reasons why this study is important is its multiethnic dimension and bilingual approach. Having in mind that the City of Skopje is a multi-ethnic environment and a city in which at the municipal level, the Macedonian or Albanian language as an official language is used, the study was conducted in both languages (AFCCQ-MK and AFCCQ-AL). Second, the results from this study can encourage the policy makers and practitioners to improve the scores in nine AFCCQ domains and the overall situation at the city and municipal level. Third, the academic community, central and local authorities, as well as the business sector can use this study as well as the validated AFCCQ-MK and AFCCQ-AL as a basis for more detailed research with larger sample at the municipal level. Fourth, with its inclusive approach, this study gives older citizens the opportunity to express their perception of age-friendliness of Skopje.

Despite the strengths, the results from this study should be interpreted with caution due to a number of limitations. First, although the sample is representative at the city level, the number of the responders on the municipal level is too small to be truly representative subsample. Second, the subgroup of older adults living in institutional care facilities were not included in this study. Third, there is a lack of qualitative data that can help in the interpretation of the results. Finally, further research is needed to investigate differences between different neighborhoods at municipal level.

## 5 Conclusions

In conclusion, according to the outcomes of the cross-culturally validated AFCCQ for use in North Macedonia for the two language groups, this study highlights that older adults experience the age-friendliness of the City of Skopje as neutral in seven domains, as “slightly dissatisfied” in one domain (*Outdoor spaces and buildings)*, and as “satisfied” in another domain (*Housing*). Despite the differences in scores in some of the AFFCQ domains between the Macedonian and Albanian communities, the two groups do not have a significant difference in the overall score on how they experience the age-friendliness of the City of Skopje. The results from this study show that older citizens are not satisfied with the situation in the city and that they face many challenges on the city and municipal level. In order to improve the situation, the central and local authorities should involve the older citizens in identifying and prioritizing their needs, as well as in the processes for creating and implementing measures and policies for improving the quality of life of older adults in the City of Skopje.

## Data availability statement

The data and supporting files are available upon request.

## Funding

This publication was initiated through COST Action CA1936 “International Interdisciplinary Network on Smart Healthy Age-friendly Environments (NET4Age Friendly)”, which is supported by 10.13039/501100000921COST (10.13039/501100000921European Cooperation in Science and Technology). This COST Action supported Prof Daniel Pavlovski with STSM grant E-COST-GRANT-CA19136-16362332.

This work was executed in conjunction with the project City&Co: Older Adults Co-Creating a Sustainable Age-friendly City (JPI project number 99950200). This project was funded by the Taskforce for Applied Research (UTC.01.1), as part of ERA-NET Cofund Urban Transformation Capacities (ENUTC), co-funded by the European Union's 10.13039/501100007601Horizon 2020 research and innovation programme under Grant Agreement No. 101003758.

The original dataset of the 2020 survey in The Netherlands was developed through funding from the Municipality of The Hague, grant number OCW/2020.1121.

## CRediT authorship contribution statement

**Daniel Pavlovski:** Writing – review & editing, Writing – original draft, Project administration, Methodology, Investigation, Funding acquisition, Formal analysis, Data curation, Conceptualization. **Jeroen Dikken:** Writing – review & editing, Writing – original draft, Methodology, Formal analysis, Conceptualization. **Elisabeta Bajrami Ollogu:** Writing – review & editing, Formal analysis. **Joost van Hoof:** Writing – review & editing, Writing – original draft, Supervision, Project administration, Methodology, Funding acquisition, Conceptualization.

## Declaration of competing interest

The authors declare that they have no known competing financial interests or personal relationships that could have appeared to influence the work reported in this paper.

## Footnotes

Appendix A Supplementary data to this article can be found online at https://doi.org/10.1016/j.heliyon.2024.e30372.
