# Supplementary material for: How older adults experience the age-friendliness of Skopje: Results of the validation of the AFCCQ for use in North Macedonia and a representative survey
Source: Heliyon. 2024 Apr 27;10(9):e30372. doi: 10.1016/j.heliyon.2024.e30372 (PMC11079086; doi:10.1016/j.heliyon.2024.e30372)
Supplement: Multimedia component 1 [file mmc1.docx]

**Appendix 1. Final version of the translated AFCCQ-MK (Macedonian)**

**ПРАШАЛНИК**

Име и презиме на анкетарот:

Датум:

Време на потполнување:

Место на потполнување:

**Пол:**

1. Машки
2. Женски

**Датум на раѓање:**

**Националност:**

1. Македонска
2. Албанска
3. Друго (која)

**Во која земја сте родени:**

**Степен на образование:**

**Во кое соседство (населба живеете):**

**Колку години живеете во градот Скопје:**

**Домот во кој што живеете е:**

1. Во ваша сопственост
2. Социјално домување
3. Под кирија
4. Друго:

**Со кого живеете:**

**Дали добивате некакви услуги во домот (чистење, лична помош):**

1. Да (од кого – да се наведе)
2. Не (да се наведе зошто)

**Дали имате хронични болести или состојби:**

1. Да
2. Не

**Дали користите бастун или количка:**

1. Да
2. Не

**Со која оценка би го оцениле Вашиот живот:**

|  |  | **ЦЕЛОСНО НЕ СЕ**  **СОГЛАСУВАМ** | **НЕ СЕ СОГЛАСУВАМ** | **НЕУТРАЛНО** | **СЕ СОГЛАСУВАМ** | **ЦЕЛОСНО СЕ СОГЛАСУВАМ** |
| --- | --- | --- | --- | --- | --- | --- |
| **1** | Лесно можам да пристапам до мојот дом | ‐2 | ‐1 | 0 | 1 | 2 |
| **2** | Луѓето кои доаѓаат да ме посетат лесно можат да пристапат до мојот дом | ‐2 | ‐1 | 0 | 1 | 2 |
| **3** | Имам доволно можности да запознаам луѓе во моето соседство | ‐2 | ‐1 | 0 | 1 | 2 |
| **4** | Активностите и настаните се организираат на места кои ми се пристапни | ‐2 | ‐1 | 0 | 1 | 2 |
| **5** | Информациите за активностите и настаните ми се доволни, а воедно и соодветни за мене | ‐2 | ‐1 | 0 | 1 | 2 |
| **6** | Сметам дека има доволно разновидни настани и активности | ‐2 | ‐1 | 0 | 1 | 2 |
| **7** | Понекогаш поради мојата возраст добивам досадни или негативни коментари | ‐2 | ‐1 | 0 | 1 | 2 |
| **8** | Понекогаш поради мојата возраст се соочувам со дискриминација | ‐2 | ‐1 | 0 | 1 | 2 |
| **9** | Јас имам доволно можности за интеракција со помладите генерации | ‐2 | ‐1 | 0 | 1 | 2 |
| **10** | Јас се чувствувам како ценет член на општеството | ‐2 | ‐1 | 0 | 1 | 2 |
| **11** | Печатените и дигиталните информации од општината и од другите социјални установи се лесни за читање од аспект на фонт и големина на буквите | ‐2 | ‐1 | 0 | 1 | 2 |
| **12** | Печатените и дигиталните информации од општината и другите социјални установи се напишани на разбирлив јазик | ‐2 | ‐1 | 0 | 1 | 2 |
| **13** | Понудата на нега и социјални услуги во мојот град е доволна за мене | ‐2 | ‐1 | 0 | 1 | 2 |
| **14** | Кога сум болен, ја добивам потребната нега и помош | ‐2 | ‐1 | 0 | 1 | 2 |
| **15** | Ако имам потреба, можам лесно да стигнам до социјалните услуги телефонски и лично | ‐2 | ‐1 | 0 | 1 | 2 |
| **16** | Имам доволно информации за социјалните услуги во моето соседство | ‐2 | ‐1 | 0 | 1 | 2 |
| **17** | Давателите на социјални услуги во моето соседство покажуваат доволно почит | ‐2 | ‐1 | 0 | 1 | 2 |
| **18** | Во мојата населба лесно може да се движи човек со дубак со тркала или со инвалидска количка | ‐2 | ‐1 | 0 | 1 | 2 |
| **19** | Во продавниците во мојата населба може лесно да се пристапи со дубак на тркала или со инвалидска количка | ‐2 | ‐1 | 0 | 1 | 2 |
| **20** | Во моето соседство лесно можам да се качам во автобус | ‐2 | ‐1 | 0 | 1 | 2 |
| **21** | На автобуските станици во моето соседство лесно може да им се пристапи и лесно се користат | ‐2 | ‐1 | 0 | 1 | 2 |
| **22** | Мојот приход ми е доволен да ги покријам основните потреби без никакви проблеми | ‐2 | ‐1 | 0 | 1 | 2 |
| **23** | Живеам добро со мојот приход | ‐2 | ‐1 | 0 | 1 | 2 |

**Appendix 2. Final version of the translated AFCCQ-AL (Albanian)**

**PYETËSOR**

**Emri dhe mbiemri i anketuesit/es:** __________________________________________________

**Data**: _____________ **Koha e realizimit:** ___________________________________________

**Vendi i realizimit**:_________________________________________________________________

| **Emri dhe mbiemri** |  | | | |
| --- | --- | --- | --- | --- |
| **Gjinia** | FEMËR | | MASHKULL | |
| **Data e lindjes:** |  | | | |
| **Përkatësia etnike** | 1. Shqiptar/e 2. Maqedonas/e | | 3. Tjetër (specifiko)______________ | |
| **Vendi ku keni lindur:** |  | | | |
| **Niveli arsimor:** |  | | | |
| **Në cilën lagje (rrethinë) jetoni:** |  | | | |
| **Prej sa vitesh jetoni në Shkup:** |  | | | |
| **Shtëpia ku jetoni është:** | 1. Në posedimin tuaj | 2. Strehim social | 3. Jetoni me qera | 4. Tjetër________ |
| **Me kë jetoni/banoni:** |  | | | |
| **A merrni ndonjë shërbim në shtëpi (pastrim, asistencë personale):** | Po (nga kush - të specifikohet): | | Jo (të speicfikohet arsyeja): | |
| **A keni sëmundje ose gjendje kronike:** | PO | | JO | |
| **A përdorni shkop/bastun ose karrige me rrota:** | PO | | JO | |
| **Si do ta vlerësonit jetën tuaj?** |  | | | |

|  | **PYETJET** | **NUK PAJTOHEM ASPAK** | **NUK PAJTOHEM** | **NEUTRAL/E** | **PAJTOHEM** | **PAJTOHEM PLOTËSISHT** |
| --- | --- | --- | --- | --- | --- | --- |
| 1 | Shtëpia ime është e qasshme për mua | -2 | -1 | 0 | 1 | 2 |
| 2 | Shtëpia ime është e qasshme për njerëzit që vijnë për të më vizituar | -2 | -1 | 0 | 1 | 2 |
| 3 | Ka mjaft mundësi për të njohur njerëz në lagjen time. | -2 | -1 | 0 | 1 | 2 |
| 4 | Aktivitetet dhe ngjarjet organizohen në vende të qasshme/aksesueshme për mua | -2 | -1 | 0 | 1 | 2 |
| 5 | Informacionet mbi aktivitetet dhe ngjarjet janë të mjaftueshme dhe të përshtatshme për mua | -2 | -1 | 0 | 1 | 2 |
| 6 | Për mendimin tim shtrirja e ngjarjeve dhe aktiviteteve është mjaft e shumëllojshme | -2 | -1 | 0 | 1 | 2 |
| 7 | Ndonjëherë më bëjnë komente të bezdisshme ose negative për shkak të moshës sime | -2 | -1 | 0 | 1 | 2 |
| 8 | Ndonjëherë më diskriminojnë për shkak të moshës | -2 | -1 | 0 | 1 | 2 |
| 9 | Kam mundësi të mjaftueshme për të ndërvepruar me gjeneratat më të reja | -2 | -1 | 0 | 1 | 2 |
| 10 | Ndihem si pjesëtar i vlerësuar i shoqërisë | -2 | -1 | 0 | 1 | 2 |
| 11 | Informatat e shtypura dhe ato digjitalet që publikon komuna apo institucionet të tjera sociale ofrohen me madhësi dhe shkrim që lexohet lehtë | -2 | -1 | 0 | 1 | 2 |
| 12 | Informatat e shtypura dhe ato digjitalet që publikon komuna apo institucionet e tjera sociale janë të shkruara në mënyrë të kuptueshme | -2 | -1 | 0 | 1 | 2 |
| 13 | Masat e kujdesit dhe shërbimeve për mirëqenie sociale në qytetin tim janë të mjaftueshme për mua | -2 | -1 | 0 | 1 | 2 |
| 14 | Kur jam i sëmurë, marr kujdesin dhe ndihmën që më nevojitet | -2 | -1 | 0 | 1 | 2 |
| 15 | Në rast nevoje, mund të arrij lehtësisht shërbimet e kujdesit dhe mirëqenies sociale nëpërmjet telefonit apo fizikisht | -2 | -1 | 0 | 1 | 2 |
| 16 | Kam informacion të mjaftueshëm mbi shërbimet e kujdesit dhe mirëqenies sociale në lagjen time | -2 | -1 | 0 | 1 | 2 |
| 17 | Punonjësit e kujdesit dhe mirëqenies sociale në lagjen time janë mjaft të respektueshëm | -2 | -1 | 0 | 1 | 2 |
| 18 | Lagja ku jetoj është e mjaft e aksesueshme për karrocat dhe mjetet e tjera me rrota | -2 | -1 | 0 | 1 | 2 |
| 19 | Dyqanet në lagjen ku jetoj janë mjaft të akesueshme për karrocat dhe mjetet e tjera me rrota | -2 | -1 | 0 | 1 | 2 |
| 20 | Unë mund të hipi lehtësisht në autobus në lagjen ku jetoj | -2 | -1 | 0 | 1 | 2 |
| 21 | Stacionet e autobusit në lagjen time janë lehtësisht të arritshme dhe të përdorshme nga unë | -2 | -1 | 0 | 1 | 2 |
| 22 | Me të ardhurat që kam i mbuloj nevojat bazike pa asnjë problem | -2 | -1 | 0 | 1 | 2 |
| 23 | Jetoj mirë me të ardhurat që kam | -2 | -1 | 0 | 1 | 2 |

**Appendix 3. The original English version of the AFCCQ-EN by Dikken et al. (2020) as used in the study in Skopje**

Name and surname of the interviewer:

Date:

Time:

Place:

**Sex:**

1. Male
2. Female

**Date of birth:**

**Nationality:**

1. Macedonian
2. Albanian
3. Other (specify)

**In which country were you born?**

**What is the highest type of education you have received? __________**

**In which neighbourhood do you live?**

**How many years have you lived in Skopje?**

**The home you live in is:**

1. In your possession

2. Social housing

3. Rented

4. Other**:**

**Do you live together or alone?**

**Do you receive care at home? (such as cleaning, personal care)?**

Yes (from whom)

No (why)

**Do you have a chronic illness or condition?**

Yes

No

**Do you use a walker or wheelchair?**

Yes

No

**What score would you like to give to your life (1 Very Low – 10 Very High)?**

|  |  | **Totally disagree** | **Disagree** | **Neutral** | **Agree** | **Totally agree** |
| --- | --- | --- | --- | --- | --- | --- |
| **1** | My house is accessible to me | ‐2 | ‐1 | 0 | 1 | 2 |
| **2** | My house is accessible to the people who come to visit me | ‐2 | ‐1 | 0 | 1 | 2 |
| **3** | There are enough opportunities to meet people in my neighbourhood | ‐2 | ‐1 | 0 | 1 | 2 |
| **4** | Activities and events are organised in places that are accessible to me | ‐2 | ‐1 | 0 | 1 | 2 |
| **5** | The information about activities and events is enough for me and also suitable for me | ‐2 | ‐1 | 0 | 1 | 2 |
| **6** | I find the range of events and activities sufficiently varied | ‐2 | ‐1 | 0 | 1 | 2 |
| **7** | I sometimes get annoying or negative remarks because of my age | ‐2 | ‐1 | 0 | 1 | 2 |
| **8** | I sometimes face discrimination because of my age | ‐2 | ‐1 | 0 | 1 | 2 |
| **9** | I have enough opportunities to interact with younger generations | ‐2 | ‐1 | 0 | 1 | 2 |
| **10** | I feel like a valued member of society | ‐2 | ‐1 | 0 | 1 | 2 |
| **11** | Printed and digital information from the municipality and other social institutions is easy to read in terms of font and size | ‐2 | ‐1 | 0 | 1 | 2 |
| **12** | Printed and digital information from the municipality and other social institutions is written in understandable language | ‐2 | ‐1 | 0 | 1 | 2 |
| **13** | The supply of care and welfare in my city is enough for me | ‐2 | ‐1 | 0 | 1 | 2 |
| **14** | When I am ill, I receive the care and help I need | ‐2 | ‐1 | 0 | 1 | 2 |
| **15** | If necessary, I can easily reach care and welfare services by telephone and in person | ‐2 | ‐1 | 0 | 1 | 2 |
| **16** | I have enough information about care and welfare services in my neighbourhood | ‐2 | ‐1 | 0 | 1 | 2 |
| **17** | Care and welfare workers in my neighbourhood are sufficiently respectful | ‐2 | ‐1 | 0 | 1 | 2 |
| **18** | My neighbourhood is sufficiently accessible for a wheeled walker or wheelchair | ‐2 | ‐1 | 0 | 1 | 2 |
| **19** | The shops in my neighbourhood are sufficiently accessible with a wheeled walker or wheelchair | ‐2 | ‐1 | 0 | 1 | 2 |
| **20** | I can easily get on the bus or tram in my neighbourhood | ‐2 | ‐1 | 0 | 1 | 2 |
| **21** | The bus and tram stops in my neighbourhood are easy to reach and use | ‐2 | ‐1 | 0 | 1 | 2 |
| **22** | My income is sufficient to cover my basic needs without any problems | ‐2 | ‐1 | 0 | 1 | 2 |
| **23** | I live well on my income | ‐2 | ‐1 | 0 | 1 | 2 |

**Appendix 4. Detailed description of Measurement Invariance (MI) analysis**

The AFCCQ is a self-report questionnaire and is used to assess how older adults feel about the age-friendliness of their respected cities. The AFCCQ consists of items that are developed to assess multiple themes and has the goal to compare groups (both in a city having different backgrounds for example, as well as cross-cultural comparison). In order to be valid for such a comparison the AFCCQ should measure identical constructs with the same structure across the different groups. When this is the case, the AFCCQ is called **measurement invariant**.

When MI does not hold, groups respond differently to the items and as a consequence factor means cannot reasonably be compared. A step-by-step description for testing MI was provided by van de Schoot et al. (2012) and was used to assess the MI of the AFCCQ-MK and AFCCQ-AL.

#### Data Preparation and Screening

Before testing invariance, it is important that the data have been properly screened. For example, if one of the groups contains more (multivariate) outliers than the other group. MI studies rely on fitting the observed covariance matrix (the data) to a model, so any bias in one of the groups due to outliers will affect factor loadings, intercepts and error variances. First, following data collection, cases with missing values will be deleted and data of both groups was properly screened for respondents with an implausible answer pattern based on Person Fit measures (Tendeiro & Tendeiro, 2018). This is important because any bias in one of the groups due to deviant answer patterns will affect factor loadings (discrimination parameter), intercepts (difficulty parameter) and error variances, which are used to assess MI (Murray, 2018). Then, the number of parameters that can be assessed with the available data are tested.

First, the **configural invariance** of the AFCCQ was tested by specifying the confirmatory factor analysis (CFA) so that it reflects how the construct was theoretically operationalised by Dikken et al. (2020). This CFA model was fitted for each language separately to test whether the same CFA was valid in each group. Next, a series of models were run for testing the level of MI using the same fit measures for assessing the fit of the increasingly constrained models (CFI/TLI, SRMR, RMSEA and χ^2^ statistics, see methods section) which should not decrease significant (∆CFI <.01 is acceptable). **Metric invariance** tests whether respondents across different groups attribute the same meaning to the latent construct under study. In the first model only the factor loadings are equal across groups (constrained) but the intercepts are allowed to differ between groups. Then, a new model is run in which only the intercepts are equal across groups (constrained), but the factor loadings are allowed to differ between groups. This tests whether the meaning of the levels of the underlying items (intercepts) are equal in both groups. **Scalar invariance** implies that the meaning of the construct (the factor loadings), and the levels of the underlying items (intercepts) are equal in both groups. If so, groups can be compared on their scores on the latent variable. To test for scalar invariance, a model will be run where the loadings and intercepts are constrained to be equal.

*References Appendix 4*

1. Dikken, J., van den Hoven, R.F.M., van Staalduinen, W.H., Hulsebosch-Janssen, L.M.T., van Hoof, J. (2020) How older people experience the age-friendliness of their city: Development of the Age-Friendly Cities and Communities Questionnaire. International Journal of Environmental Research and Public Health 17(18):6867. doi:10.3390/ijerph17186867
2. van de Schoot, R., Lugtig, P., Hox, J. (2012) A checklist for testing measurement invariance. European Journal of Developmental Psychology 9(4):486–492. https://doi.org/10.1080/17405629.2012.686740
3. Tendeiro, J.N., Tendeiro, M.J.N. (2018) Package ‘PerFit’. CRAN, CRAN.
4. Murray, J.S. (2018) Multiple Imputation: A Review of Practical and Theoretical Findings. Statistical Science 33(2):42-159. doi: 10.1214/18-STS644
